# Proteoglycan serglycin promotes non-small cell lung cancer cell migration through the interaction of its glycosaminoglycans with CD44

**DOI:** 10.1186/s12929-019-0600-3

**Published:** 2020-01-02

**Authors:** Jing-You Guo, Chu-Hsuan Chiu, Mei-Jung Wang, Fu-An Li, Jeou-Yuan Chen

**Affiliations:** 1 Institute of Biomedical Sciences, Academia Sinica, 128 Academia Road, Section 2, Taipei, 115 Taiwan; 2 Department of Life Sciences and Institute of Genome Sciences, National Yang-Ming University, Taipei, Taiwan, Republic of China

**Keywords:** CD44, Cell migration, Chondroitin sulfate glycosaminoglycan, Proteoglycan, Serglycin, Glycoaminoglycans, Cytoskeleton, SRC

## Abstract

**Background:**

Serglycin (SRGN), previously recognized as an intracellular proteoglycan involved in the storage processes of secretory granules, has recently been shown to be upregulated in several solid tumors. We have previously shown that SRGN in non-small cell lung cancer (NSCLC) promotes malignant phenotypes in a CD44-dependent manner and increased expression of SRGN predicts poor prognosis of primary lung adenocarcinomas. However, the underlying mechanism remains to be defined.

**Methods:**

Overexpression, knockdown and knockout approaches were performed to assess the role of SRGN in cell motility using wound healing and Boyden chamber migration assays. SRGN devoid of glycosaminoglycan (GAG) modification was produced by site-directed mutagenesis or chondroitinase treatment. Liquid chromatography/tandem mass spectrometry was applied for quantitative analysis of the disaccharide compositions and sulfation extent of SRGN GAGs. Western blot and co-immunoprecipitation analyses were performed to determine the expression and interaction of proteins of interest. Actin cytoskeleton organization was monitored by immunofluorescence staining.

**Results:**

SRGN expressed by NSCLC cells is readily secreted to the extracellular matrix in a heavily glycosylated form attached with mainly chondroitin sulfate (CS)-GAG chains, and to a lesser extent with heparin sulfate (HS). The CS-GAG moiety serves as the structural motif for SRGN binding to tumor cell surface CD44 and promotes cell migration. SRGN devoid of CS-GAG modification fails to interact with CD44 and has lost the ability to promote cell migration. SRGN/CD44 interaction promotes focal adhesion turnover via Src-mediated paxillin phosphorylation and disassembly of paxillin/FAK adhesion complex, facilitating cell migration. In support, depletion of Src activity or removal of CS-GAGs efficiently blocks SRGN-mediated Src activation and cell migration. SRGN also promotes cell migration via inducing cytoskeleton reorganization mediated through RAC1 and CDC42 activation accompanied with increased lamellipodia and filopodia formation.

**Conclusions:**

Proteoglycan SRGN promotes NSCLC cell migration via the binding of its GAG motif to CD44. SRGN/CD44 interaction induces Rho-family GTPase-mediated cytoskeleton reorganization and facilitates Src-mediated focal adhesion turnover, leading to increased cell migration. These findings suggest that targeting specific glycans in tumor microenvironment that serve as ligands for oncogenic pathways may be a potential strategy for cancer therapy.

## Background

Glycans, existing as membrane-bound or intracellular glycoconjugates or as secreted molecules embedded in the extracellular matrix (ECM), are involved in various pathophysiological steps of tumor formation and progression [1]. Glycosaminoglycans (GAGs) are linear polysaccharides comprised of repeating disaccharide units present in free form as hyaluronic acid or covalently attached to a protein backbone expressed as proteoglycans (PGs) such as heparin/heparan sulfate (HS), chondroitin sulfate (CS)/dermatan sulfate (DS), and keratan sulfate (KS) [2]. Through interactions with extracellular ligands, cell surface receptors, and extracellular matrix components, GAGs and PGs regulate a wide range of cellular processes, including infection, blood coagulation, angiogenesis and axonal regeneration. PGs and GAGs are frequently overrepresented in human cancers, and engaged in cell signaling regulating cell proliferation, cell adhesion, and cell motility [3].

Both HS and CS are sulfated GAGs, consisting of repeating disaccharide units of glucuronic acid (GlcA) and N-acetylglucosamine (GlcNAc) (for HS) or N-acetylgalactosamine (GalNAc) (for CS), that attach to specific serine residues of the core protein through a common linker region (GlcAβ1-3Galβ1-3Galβ1-4Xylβ1-) [4]. GAGs are capable of interacting with a number of important growth factors and functional proteins. HS has been shown to interact with growth factors including fibroblast growth factors (FGFs), hepatocyte growth factor (HGF), insulin-like growth factors (IGFs) and transforming growth factor β (TGFβ), and promotes cancer cells adhesion, migration/invasion and angiogenesis [2]. CS of different sulfation patterns are shown to interact with different growth factors and enzymes involved in ECM degradation to facilitate cancer cells migration and invasion [2, 5]. Interestingly, HS and CS proteoglycans have been demonstrated to regulate numerous cell surface signaling events with opposite effects on cell function in various pathological settings [6, 7].

Serglycin (SRGN) was initially identified as a secretary granule proteoglycan functioning in promoting secretory granule storage processes first in mast cells [8], then in many other hematopoietic cells, endothelial cells and nasopharyngeal carcinomas [9]. SRGN has also been reported to be secreted from secretory granules and serve as a novel ligand for CD44 to regulate lymphoid cell adherence and activation [10]. We have previously shown that SRGN is frequently overexpressed in lung adenocarcinomas, and functions in promoting NSCLC cell migration, invasion and stemness in a CD44-dependent manner [11]. SRGN has also been identified as the topmost differentially enriched protein in plasma-derived extracellular vesicles in lung adenocarcinoma patients, suggesting a potential role of SRGN to serve as a biomarker for lung cancer [12]. CD44, the principle cell surface receptor for hyaluronan (HA), has been reported as a cancer stem cell signature in many cancers of hematopoietic and epithelial origins [13]. Via interacting with various ligands, CD44 functions as an adhesion molecule for cell-cell and cell-ECM interactions. HA binding to CD44 promotes cancer cell migration through SRC-induced cortactin cytoskeleton function [14]. SRGN has been reported to bind to CD44 closed to the extracellular HA binding domain [10]. However, till now, the molecular mechanism of SRGN/CD44-elicited migration is still unclear.

SRGN expressed by different cells have been shown to be covalently attached by GAGs of different types of CS/HS modifications and sulfation codes [9, 15]. In connective type mast cells, SRGN is modified by heparin, which is characterized with a high extent of sulfation. Interestingly, SRGN has also been shown to be decorated with high sulfated CS in mucosal type and bone marrow-derived mast cells, activated monocytes and macrophages. Increasing evidence has indicated that different disaccharide compositions and extent of sulfation may have great effects on the binding and biological activity of GAGs. To further investigate SRGN-elicited malignant phenotypes in NSCLC cells, in this study, we defined the compositions and sulfation patterns of GAGs in NSCLC-derived SRGN and showed that NSCLC-derived SRGN is heavily decorated with CS, and these CS-GAGs are engaged in CD44 binding and drive SRGN/CD44-elicited cell motility. We further showed that SRGN promotes NSCLC cell migration via RAC1/CDC42-induced cytoskeleton reorganization and SRC-mediated focal adhesion turnover.

## Materials and methods

### Cell culture and antibodies

Human non-small cell lung cancer (NSCLC) H460 and H1299 cells, and duodenal adenocarcinoma HuTu80 (previously named HTB-40) cells were cultured in RPMI-1640 medium supplemented with 10% FBS and 1% penicillin/streptomycin, and maintained at 37 °C in humid air with 5% CO_2_ condition. Cell lines were tested routinely to confirm the absence of mycoplasma, and have been recently authenticated by short tandem repeat (STR) profiling (Bioresource Collection and Research Center (BCRC) of Food Industry Research and Development Institute, Taiwan). The hybridoma for Hermes-3 (H-3) anti-CD44 antibody was from the American Type Culture Collection (ATCC, Manassas, VA, USA). Antibodies against p-Src (Y416) (Cell Signaling Technology, Danvers, MA, USA), Src (Cell Signaling Technology), p-ERK1/2 (Cell Signaling Technology), ERK2 (Santa Cruz Biotechnology, Santa Cruz, CA, USA), p-paxillin (Y118) (Biosource International, Camarillo, CA, USA), paxillin (Santa Cruz Biotechnology), FAK (BD Biosciences, San Jose, CA, USA) were used for immunoprecipitation and Western blot in this study. Unsaturated disaccharide standards of ∆CS-0S [∆UA-GalNAc], ∆CS-4S [∆UA-GalNAc(4S)], ∆CS-6S [∆UA-GalNAc(6S)], ∆CS-4S6S [∆UA-GalNAc(4S6S)], ∆HS-0S [∆UA-GlcNAc], ∆HS-6S [∆UA-GlcNAc(6S)], ∆HS-NS [∆UA-GlcNS], ∆HS-2SNS [∆UA(2S)-Glc(NS)], ∆HS-NS6S [∆UA-GlcNS(6S)], and ∆HS-tS [∆UA(2S)-GlcNS(6S)], where ∆UA is β-D-glucuronic acid, GalNAc is β-D-N-acetylgalactosamine, GlcNAc is α-D-N-acetylglucosamine GlcNS is α-D-N-sulfoglucosamine and S is sulfo) were purchased from Iduron (Macclesfield, UK).

### Constructs and establishment of stable cell clones

SRGN cDNA was generated by polymerase chain reaction (PCR) using cDNA derived from 293 T cells as template and following SRGN primers (Forward 5′-CGA TGA ATT CAT GAT GCA GAA GCT ACT CAA ATG-3′ and Reverse 5′-CGA TGG ATC CTA ACA TAA AAT CCT CTT CTA ATC-3′). The PCR product was subcloned into p3xFLAG-CMV-13 vector (Sigma-Aldrich, St. Louis, MO, USA) using EcoRI and BamHI sites, followed by sequence confirmation. To generate SRGN(S/A) mutant cDNA, two-step PCR was performed using p3xFLAG-CMV-13-SRGN as template. In the first step, two reactions were performed using two sets of primers, respectively. Reaction 1 was performed using the SRGN forward primer and a reverse primer containing sequences corresponding to S/A mutation (5′-GCC AGC CCC AGC TCC TGC TCC AGC GCC GGC GCC GGC GCC GAA GCC TGC TCC AGC GTA GTC CTC AGA AAG TGG-3′) to generate SRGN(S/A) cDNA fragment 1. Reaction 2 was performed using forward primer containing sequences corresponding to S/A mutation (5′-TAC GCT GGA GCA GGC TTC GGC GCC GGC GCC GGC GCT GGA GCA GGA GCT GGG GCT GGC TTC CTA ACG GAA ATG-3′) and SRGN reverse primer to generated SRGN (S/A) cDNA fragment 2. In the 2nd-step PCR, the SRGN(S/A) cDNA fragments 1 and 2 were mixed and used as template, and PCR was performed using the SRGN forward and reverse primers to generate a full length SRGN (S/A) cDNA. The PCR product was subcloned into p3xFLAG-CMV-13 vector, followed by sequence confirmation. H1299 cells were transfected with p3xFLAG-CMV-13-SRGN and p3xFLAG-CMV-13 plasmids, respectively, by LF2000 (Invitrogen, Dublin, Ireland), and cultured in medium containing G418 (800 μg/ml, Sigma-Aldrich) for 14 days, yielding H1299/SRGN and H1299/SRGN(S/A) cell clones. The expression vector encoding the full-length standard form of CD44 was constructed by PCR amplification using human placenta cDNA as a template in the presence of CD44 primers (Forward 5′-AAG CTT GAC ACG ATG GAC AAG TTT TG-3′ and Reverse 5′-GAA TTC ATA ATG ATG TAG GTG TAA CAC-3′), followed by subcloning the PCR product into pcDNA 3.1/myc-His-A vector (Invitrogen) at XhoI and KpnI restricted sites, followed by sequence confirmation. To generate CD44-expressing clones, HuTu80 cells were transfected with CD44 expression vector using LF2000, and cultured in medium containing G418 (500 μg/ml) for 2 weeks. CD44-expressing HuTu80 cells were further sorted by flow cytometry using CD44-FITC antibody (clone G44–26, BD Biosciences). The resultant CD44-negative (HuTu80/Mock) and CD44-positive (HuTu80/CD44) cells were maintained in medium containing G418 (500 μg/ml). Knockdown of SRGN in H460 cells by lentivirus-shRNA has been previously described [11]. SRGN-KD cells were maintained in medium containing puromycin (1 μg/ml, Sigma-Aldrich). CD44 was knocked out in H1299 cells using bacterial CRISPR/Cas9 system. In brief, CD44 sgRNA (5′-GCG CCA GGC TCA GCG GCA CG-3′), purchased from the National RNAi Core Facility, Taiwan, was constructed into the all-in-one sgRNA/CAS9 expression lentivector and Lentivirus-based knock-out of CD44 was performed following the online protocols (http://rnai.genmed.sinica.edu.tw/webContent/web/protocols) from the Core Unit. Single clones were selected and confirmed as CD44-null cell clones, and maintained in puromycin-containing medium.

### Conditioned medium (CM) preparation and enzyme digestion of GAG chains

H1299/Mock, H1299/SRGN and H1299/SRGN (S/A) cells as well as H460/Sh-CTRL and H460/Sh-SRGN cells were seeded and cultured in 150-mm dishes. When cell density reaching 90% confluence, cells were washed with PBS twice and replenished with 20 ml of serum-free RPMI-1640 medium, followed by further incubation for 48 h. The CM was collected and filtered through 0.45 μm filter, concentrated, and desalted with ddH_2_O twice to a final volume of 100 μl by using Amicon Ultra-15 Centrifugal Filter Unit (UFC901024, EMD Millipore, Burlington, MA, USA) at 14,300×*g* centrifugation. Protein concentration in the concentrated CM was assessed by Bradford Protein Assay (BIO-RAD Life Science, Hercules, CA, USA). To digest SRGN GAG chains, an aliquot of CM that was measured to contain 75 μg protein was treated with 100 mU of Chondroitinase (Chase) B (Sigma-Aldrich), 100 mU of ChaseAC (Sigma-Aldrich), 100 mU of ChaseABC (Sigma-Aldrich), or 100 mU of Heparinase I + III (Sigma-Aldrich) for 24 h at 37 °C, followed by western blot analysis using designated antibodies, including anti-SRGN (HPA000759, Sigma-Aldrich), anti-HS (amsbio LLC, Cambridge, MA, USA), anti-∆HS stub (amsbio LLC), anti-CS (Abcam, Cambridge, UK), anti-∆C4S stub (Sigma-Aldrich) and anti-∆C6S stub (LifeSpan Biosciences, Seattle, WA, USA).

### GAG purification and high performance liquid chromatography–tandem mass spectrometry (LC-MS/MS) analysis of GAG disaccharide units

CM was prepared and concentrated as described above. Protein concentration was determined using Bradford reagent. For GAG purification, an aliquot of CM that was measured to contain 250 μg protein was mixed with 100 μl of actinase E (20 mg/ml), with ddH_2_O added to a final volume of 600 μl, and incubated at 55 °C for 24 h. After heat inactivation at 100 °C for 10 min, the reaction mixture was centrifuged at 10,000×*g* for 10 min at 4 °C. The supernatant was collected and pellet was re-suspended in 50 μl of ddH_2_O and centrifuged at 10,000×*g* for 10 min at 4 °C to collect the supernatant. The supernatants were combined, mixed with 200 μl of Urea buffer (8 M urea, 2% CHAPS, pH 8.3), and loaded onto a Vivapure MiniQ H spin column (#VS-1X01QH24, Sartorius Corporate, Goettingen, Germany) pre-equilibrated with the urea buffer. After spinning at 2000×*g* for 5 min at 4 °C, the flow-through was collected and re-loaded to the same column for spinning. These procedures were repeated for two more times. The column was washed by 400 μl of wash buffer (200 mM NaCl) by spinning at 2000×*g* for 5 min at 4 °C, and eluted by 400 μl of elution buffer (2.74 M NaCl) by spinning. The elution step was repeated for two more time. The eluents were combined (~ 1.2 ml) and concentrated to a volume of 50 μl by an Amicon Ultra-0.5 Centrifugal Filter Unit (#UFC500396, 3 kDa, Millipore) centrifuged at 14,300×*g* at 4 °C, and desalted by mixing with 450 μl of ddH_2_O followed by centrifugation for six times. The desalted GAGs sample was then treated with 100 mU of ChaseABC and 100 mU of Heparinase I + III for 24 h at 37 °C. The GAG samples were lyophilized and disaccharides were subjected to fluorescence labeling with 2-aminoacridone (AMAC). The freeze-dried disaccharides (2 μg) was added in 10 μl 2-aminoacridone (AMAC) solution (100 mM AMAC in glacial acetic acid/dimethyl sulfoxide (DMSO), 3:17 v/v) and incubated at room temperature for 15 min. Then, 10 μl of 1 M NaBH_3_CN was added to the reaction mixture and incubated at 45 °C for 4 h. The samples were lyophilized and ready for LC-MS/MS analysis [16]. For standard curve, the AMAC-labeled disaccharides standards mixture (sodium salt of 10 unsaturated CS/DS disaccharides; Iduron, UK) was diluted to series concentrations (1–27 ng/ul) in 50% (v/v) aqueous DMSO.

LC-MS/MS analysis was performed by an ACQUITY UPLC I-Class System (Milford, MA, USA) coupled with a mass spectrometer (Orbitrap Elite, Thermo Scientific, Waltham, MA, USA) equipped with heated electrospray ionization (HESI) source. The derivatized samples are separated by gradient elution on a C18 column (BEH C18 column, 2.1 mm × 150 mm, 1.7 μm, Waters, Milford, MA, USA) at a flow rate of 200 μl/min. The column temperature was maintained at 25 °C. The mobile phases consisted of 80 mM ammonium acetate (eluent A) and 100% methanol (eluent B). The gradient program was linearly changed as follows: 12% B from 0 to 5 min, 12–14% B from 5 to 15 min, 14% B from 15 to 17.5 min, 14–30% B from 17.5 to 32.5 min, 30–100% B from 32.5 to 62.5 min. Mass spectra were acquired in Negative ion mode for MS and MS/MS function. Quantification analysis of AMAC-labeled disaccharides was performed based on Standard Curve method. The linearity is evaluated by the correlation coefficient (r^2^). The absolute amount of disaccharides in samples was calculated based on the equation of standard curve. Data acquisition and peak area integration were accomplished by using Xcalibur 2.2 software (Thermo Fisher Scientific). Standard calibration curves were generated with three technical replicates of five concentrations and used for determination of the absolute amount of the individual HS and CS disaccharides.

### Cell migration assay and immunofluorescence of actin cytoskeleton reorganization

For Boyden chamber migration assay, 2 × 10^6^ cells were seeded in 60-mm dish and incubated overnight. Cells were then incubated in serum-free medium for 24 h, and suspended by enzyme-free Gibco Cell Dissociation Buffer (Thermo Fisher Scientific), washed by complete cultured medium once, washed by PBS twice, re-suspended in RPMI medium containing 0.1% FBS, and subjected to migration assay using the standard 48-well chemotaxis chamber as previously described [17]. Briefly, cells were seeded in the upper chamber (3 × 10^3^ cells/well in 50 μl of medium containing 0.1% FBS), with lower chamber loaded with medium containing 5% FBS. In some experiments, the lower chamber was loaded with medium containing 2% FBS as specified. The lower surface of the membrane (#K80SH58050, GE Healthcare Life Sciences, Pittsburgh, PA, USA) inserted between the two chambers was coated with 0.1 μg/μl of fibronectin (Millipore). After 3 or 5 h as specified, the cells migrated through the insert and attached on the lower surface of the insert were stained with 1 μg/ml of Hoechst 33258, and counted by ImageJ. In some experiments, cells were re-suspended in the CM that was pretreated with or without ChaseABC and loaded into the upper chamber for migration assay. To inhibit Src activity, cells were incubated in serum-free medium for 24 h, and incubated in medium containing 10 μM of PP2 for 3 h, followed by Boyden chamber migration assay.

For wound healing assay, 1 × 10^4^ cells were seeded into the Culture-Insert 2 Well (ibidi Gmbh, Grafelfing, Germany) and incubated overnight. Following cell attachment, inserts were removed and cells were washed with PBS twice. H1299 cells were cultured in serum-free RPMI medium and H460 cells were cultured in 2% FBS-containing RPMI medium for 72 h. Cell migration was observed under microscope at 24-h intervals. To observe F-actin cytoskeleton reorganization during the process of wound closuring, cells were seeded into the Culture-Insert 2 Well on slides, and allowed cells to migrate for a designated time (24 h for H1299 cells and 72 h for H460 cells), followed by cell fixation in 4% paraformaldehyde for 30 min. Cells were washed with Tris-Buffered Saline (TBS, 50 mM Tris-Cl, pH 7.6; 150 mM NaCl) three times and blocked with TBS containing 0.1% Triton X-100 and 3% bovine serum albumin (BSA) overnight at 4 °C. F-actin was stained by Alexa Fluor™ 594 Phalloidin (Invitrogen) and cells were counterstained with 4′,6-diamidino-2-phenylindole dihydrochloride (DAPI) and observed under a laser-scanning confocal system (MRC 1000; Bio-Rad). Since overexpression and knockdown of SRGN had little effects on cell proliferation, the measurements of cell migration and wound closure were not corrected by cell proliferation.

### Rho GTPase activation assay

5 × 10^6^ cells were seeded in serum-free medium on FN-coated dishes and incubated for 30 min. Cells were lysed and assayed for Rac1 and Cdc42 activation using Rac/cdc42 Activation Assay Kit (Upstate Biotechnology, Lake Placid, NY, USA) and RhoA activation using RhoA Activation Assay (Upstate Biotechnology). Briefly, cells were lysed in 1X MLB lysis buffer containing 10 μg/ml leupeptin, 10 μg/ml aprotinin, 1 mM NaF and 1 mM Na_3_VO_4_. Two mg of cell lysate was mixed with 20 μg of PAK-1 PBD agarose and 0.3 mg of cell lysate was mixed with 50 μg of Rhotekin-RBD agarose, and the reaction mixtures were rocked gently at 4 °C for 60 min. Agarose was collected by centrifugation at 14,000×*g* for 5 s, and washed with 1X MLB three times. Agarose was resuspended in 20 μl of 2X Laemmli reducing sample buffer and boiled for 5 min. The supernatant and agarose pellet were mixed and subjected to Western blot analysis. Rac1-GTP, Cdc42-GTP and RhoA-GTP pulled down by agarose beads were detected using specific antibodies.

### CD44-Fc pulldown assay

Cells were seeded onto four 150-mm dishes and CM was prepared after cells were cultured in serum-free medium for 48 h as described above. The CM was concentrated and mixed with 1 μg of human CD44-Fc recombinant fusion protein (R&D Systems, Minneapolis, MN, USA) or IgG control and rocked gently in a rotating shaker at 37 °C for 18 h. The reaction mixture was mixed with 20 μl of magnetic beads reagent (GE Healthcare Life Sciences, Pittsburgh, PA, USA,) and incubated at 37 °C for 2 h. Magnetic beads were collected by magnetic stand and supernatant discarded. The beads were washed with wash buffer (25 mM Tris-HCl, pH 7.6, 200 mM NaCl, 0.1% NP-40, 6% glycerol, 1 mM EDTA and 0.5 mM DTT) for three times, and suspended in 20 μl of 2X Laemmli reducing sample buffer and boiled for 5 min, followed by western blot analysis using anti-SRGN antibody.

### Statistical analysis

Data were presented as mean ± s.d. from at least three independent experiments. Two-tailed Student’s t test was employed. *P* < 0.05 was considered to be statistically significant. *, *P* < 0.05; **, *P* < 0.01 and ***, *P* < 0.001.

## Results

### SRGN promotes lung cancer cell migration in a CD44-dependent manner

**Fig. 1:**
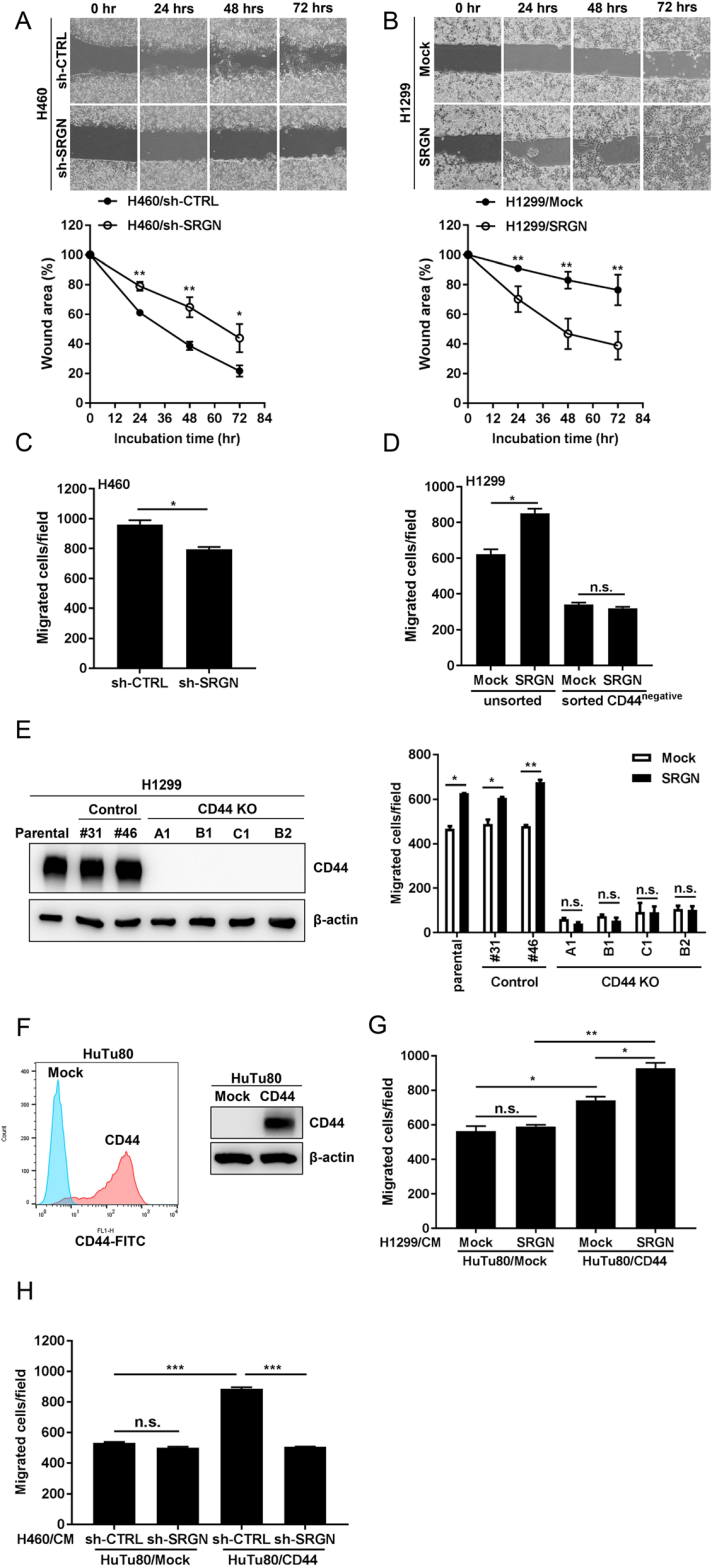
SRGN promotes NSCLC cell migration in a CD44-dependent manner**. a-b** Wound healing assay was performed by seeding NSCLC-H460 (**a**) and -H1299 (**b**) cells in the Culture-Insert 2 Well μ-Plates. After removing the insert, wound closure was determined at 0, 24, 48, and 72 h. Wound area was assessed by ImageJ and normalized with respect to the area at 0 h. **c** H460/sh-CTRL and H460/sh-SRGN cells were cultured in serum-free medium for 24 h and subjected to Boyden chamber migration assay. Migrated cells were counted in 3 h. **d** H1299/Mock and H1299/SRGN cells were sorted based on CD44 expression. The unsorted as well as CD44-negative H1299/Mock and H1299/SRGN cells were cultured in serum-free medium for 24 h, and subjected to Boyden chamber migration assay. The migrated cells were counted in 3 h. **e** The parental H1299 cells and the Control-KO and CD44-KO cell clones generated using the CRISPR/Cas9 system were subjected to Boyden chamber migration assay as described above. The expression of CD44 was shown by western blot. **f** CD44 expression in HuTu80/Mock and HuTu80/CD44 cells was assessed by flow cytometry (left panel) and western blot (right panel). **g-h** HuTu80/Mock and HuTu80/CD44 cells were suspended in conditioned media derived from H1299/Mock and H1299/SRGN cells (**g**) as well as from H460/sh-CTRL and H460/sh-SRGN cells (**h**), and subjected to Boyden chamber migration assay as described above. The Boyden chamber assays were performed by loading the lower chamber with medium containing 5% FBS in (**c**) and (**h**), and 2% FBS in (**d**), (**e**) and (**g**). Data are presented as the mean ± SD of three independent experiments. *P < 0.05, **P < 0.01 and ***P < 0.001 by Student’s *t*-test

We have previously shown that SRGN is frequently overexpressed in lung cancer and its upregulated expression correlates to poor prognosis [11]. In this study, we further examined the mechanisms underlying SRGN-mediated functions using gain- and loss-of-function approaches. Since NSCLC-H460 cells express SRGN at high level, we knocked down the expression of endogenous SRGN in H460 cells and examined the biological outcome. As shown in Fig. 1a, knockdown (KD) of the expression of SRGN greatly delayed the process of wound closure in H460 cells. In contrast, overexpression of SRGN in NSCLC-H1299 cells, that express endogenous SRGN at undetectable level, significantly accelerated wound healing (Fig. 1b). Boyden chamber migration assay further confirmed that KD of SRGN in H460 cells suppressed cell migration (Fig. 1c), and overexpression of SRGN in H1299 cells greatly enhanced cell migration (Fig. 1d). SRGN was initially demonstrated as a novel ligand for cell surface receptor CD44 [10]. We have previously shown that CD44 is required for SRGN-elicited malignant phenotypes in NSCLC cells [11]. In consistence, we showed that SRGN-mediated migration was subverted in H1299 cells that were sorted to be negative for CD44 expression (Fig. 1d). To further support the role of CD44 in SRGN-mediated migration, we generated H1299 cell clones in which *CD44* gene has been knocked out using CRISPR/Cas9 technology. As shown in Fig. 1e, knockout of *CD44* gene greatly impaired H1299 cell migration. Most importantly, H1299 cell clones from which *CD44* gene was knocked out were resistant to SRGN-mediated cell migration. SRGN is commonly regarded as an intracellular proteoglycan that is present in the secretory granules in a wide range of hematopoietic cells, including mast cells and neutrophils [18]. However, SRGN has also been shown to be constitutively secreted by macrophages, multiple myeloma cells and nasopharyngeal carcinoma [19–21]. Because the exact function of SRGN has been shown to be varied to a large extent under different cellular context [9], we further examined the expression pattern of SRGN in NSCLC cells. As shown in Additional file 1: Figure S1, SRGN expressed by NSCLC cells was readily secreted to the extracellular space as a macromolecule with extensive post-translational modifications. To further support an indispensable role of CD44 in mediating SRGN-elicited function, we collected SRGN-containing conditioned medium from H1299/SRGN and H460/Sh-CTRL cells and incubated the conditioned medium with human duodenum adenocarcinoma HuTu80 cells in which the expression of CD44 was undetectable and HuTu80 cell clones stably expressing ectopically introduced CD44. As shown, SRGN-containing medium promoted the migration of HuTu80 cells that expressed ectopically introduced CD44, but has no effect on mock-transfected HuTu80 cells (Fig. 1f and g). These findings demonstrate that SRGN promotes NSCLC cell migration in a CD44-dependent manner.

### SRGN is secreted by NSCLC cells in a glycosylated form covalently linked with sulfated glucosaminoglycan chains

**Fig. 2:**
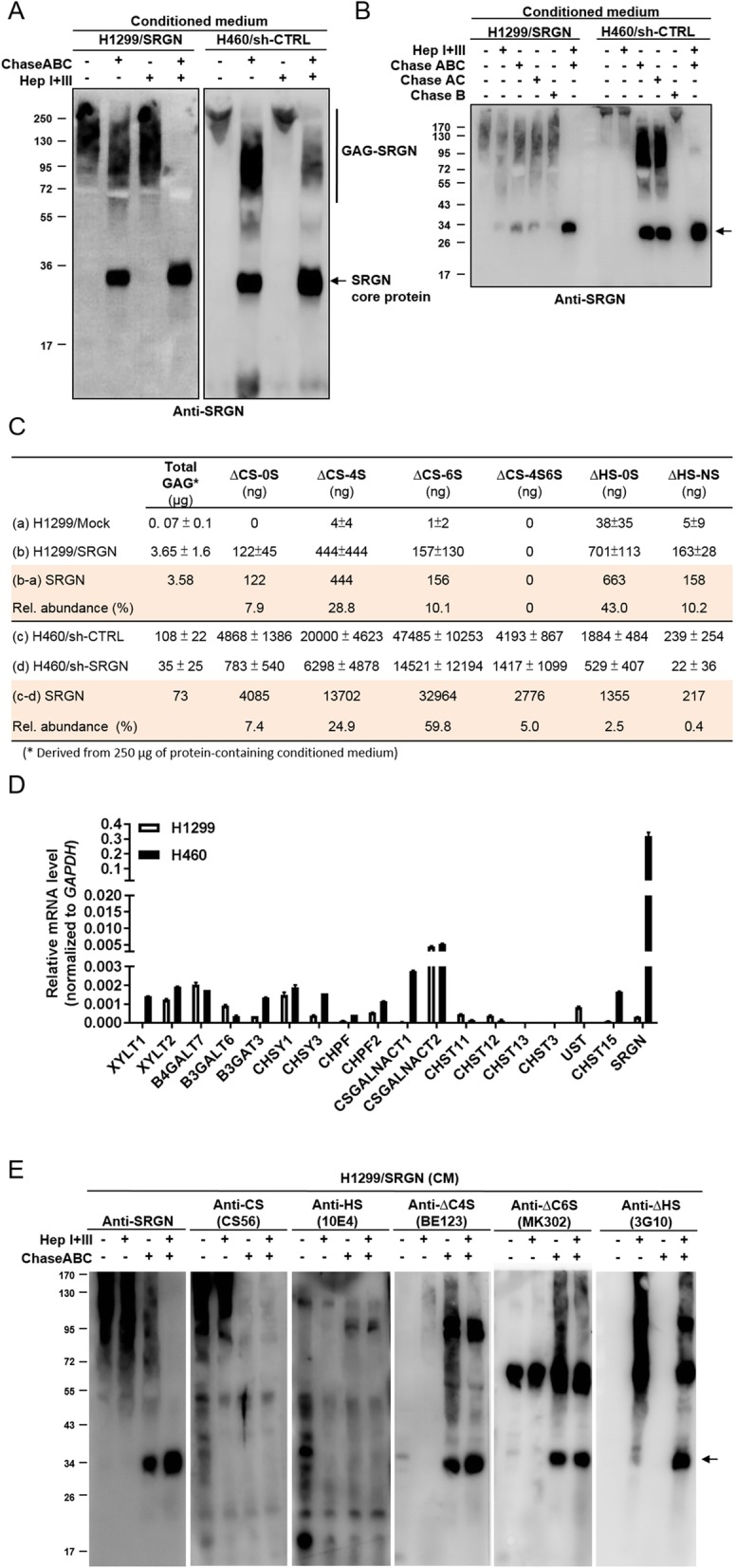
NSCLC cells secret SRGN heavily linked with CS-GAG side chains. **a-b** CM derived from designated cells was treated with 100 mU of enzymes as indicated for 24 h at 37 °C, followed by western blot analysis against anti-SRGN antibody. **c** Absolute amounts of GAGs and GAG-derived disaccharides in the CM of H1299/Mock versus H1299/SRGN cells and H460/Sh-CTRL versus H460/Sh-SRGN cells were quantitated. An aliquot of CM that was measured to contain 250 μg protein was used for GAG extraction. Total GAG amount was assessed by blyscan glycosaminoglycan assay kit. Crude GAGs were digested by ChaseABC (100 mU) and Heparanase I + III (100 mU) for 24 h at 37 °C, and subjected to LC-MS/MS analysis of CS and HS disaccharides. Absolute quantification was conducted by constructing standard curves for the individual disaccharide standard products. The amounts of GAGs and disaccharides in relation to altered expression of SRGN were obtained by normalizing the amounts of GAGs and individual disaccharides in H1299/SRGN and H460/sh-CTRL to those in H1299/Mock and H460/sh-SRGN cells, respectively. **d** RT-PCR analysis of the expression of SRGN, HS and CS synthesis genes in H1299 and H460 cells. Relative expression is shown by comparing the expression of each individual gene to that of GAPDH. **e** CM derived from H1299/SRGN cells was treated with or without enzymes as indicated for 24 h at 37 °C, followed by western blot analysis. Secretory proteins carrying compact HS and CS chains were assessed by anti-HS (10E4) and anti-CS (CS56) antibodies, respectively. SRGN core protein carrying ∆HS, ∆C4S and ∆C6S stubs was determined by anti-∆HS (3G10), anti-∆C4S (BE123) and anti-∆C6S (MK302) antibodies

Other than hematopoietic malignancies [19], several solid tumors were reported to overexpress SRGN, and increased expression of SRGN correlated with aggressive phenotypes in cancers of lung, breast, and nasopharynx [11, 21, 22]. When expressed by different types of cells, SRGN was shown to display structural diversity for being glycosylated with variable types of glycosaminoglycans (GAGs) [9, 23]. Here, we examined the GAG modifications of endogenous SRGN expressed by NSCLC-H460 cells. SRGN-containing conditioned medium was collected for enzymatic digestion. As shown in Fig. 2a, chondroitinase ABC (ChaseABC) treatment which degrades the CS/DS chains yielded a substantial amount of SRGN core protein. On the other hand, SRGN was less susceptible to the digestion by Heparinases I + III, which degrades HS-GAG. When digested by Chondroitinase ABC and Heparinase I + III together, SRGN was converted to the size of its core protein (Fig. 2a). Similar patterns were observed with SRGN ectopically expressed in H1299 cells. These data suggested that SRGN secreted from NSCLC cells was attached with mainly elongated CS/DS- and to a lesser degree HS-GAG chains. When SRGN was further subjected to enzymatic digestion using different chondroitinases, SRGN was shown to be more susceptible to chondroitinases AC than chondroitinase B, suggesting that SRGN was modified to a higher degree by CS-GAG than DS-GAG (Fig. 2b).

The sulfation code of GAG has been demonstrated to be important for its biological function [4]. We further quantitatively determined the relative amounts of GAGs and the constituent disaccharides in SRGN expressed by NSCLC cells. In H1299 cells, overexpression of SRGN significantly increased the amount of GAGs in the CM to almost 50 fold. KD of SRGN in H460 cells reduced approximately 70% of the GAGs in the CM. These data suggest that SRGN was the predominant proteoglycan present in the CM of H1299/SRGN and H460/sh-CTRL cells (Fig. 2c). The amount of GAGs in the CM of H460 cells was exceedingly higher than that derived from H1299 cells. In consistence, RT-PCR analysis also showed that *SRGN* transcripts were expressed at significantly higher level in H460 as compared to that in H1299 cells (Fig. 2d). Interestingly, transcripts of *XYLT1* and *CSGALNACT1*, which are involved in the initiation of the tetrasaccharide linkage and CS chain, respectively, were also expressed at significantly higher levels in H460 cells (Fig. 2d). For assessing the relative amounts of disaccharide components, CM-derived GAGs were subjected to enzymatic digestion followed by high performance liquid chromatography (HPLC)-tandem mass spectrometry. Absolute quantification was conducted by constructing a standard curve for the individual ∆CS-0S [∆UA-GalNAc], ∆CS-4S [∆UA-GalNAc(4S)], ∆CS-6S [∆UA-GalNAc(6S)], ∆CS-4S6S [∆UA-GalNAc(4S6S)], ∆HS-0S [∆UA-GlcNAc], ∆HS-6S [∆UA-GlcNAc(6S)], ∆HS-NS [∆UA-GlcNS], ∆HS-2SNS [∆UA(2S)-Glc(NS)], ∆HS-NS6S [∆UA-GlcNS(6S)], and ∆HS-tS [∆UA(2S)-GlcNS(6S)] disaccharide standard products. Additional file 2: Figure S2A shows the elution profiles of CS- and HS-derived disaccharide standards by HPLC, along with tandem mass analysis for identification of the signature fragments. Additional file 2: Figure S2B shows LC-MS/MS analysis of the constituent disaccharides of GAGs related to altered expression of SRGN in NSCLC cells, which revealed high levels of ∆CS-0S, ∆CS-4S, ∆CS-6S, ∆HS-0S and ∆HS-NS disaccharides related to SRGN expressed by both H1299/SRGN and H460/sh-CTRL cells. Notably, HS-0S and HS-NS were readily detected among the six HS-derived disaccharides, and others were detected at much lower levels. Taken together, these results showed that SRGN derived from NSCLC cells was heavily decorated with sulfated GAGs, with CS as the predominant species. Western blot analysis of SRGN using antibodies recognizing the compact elongated CS- and HS-GAG chains confirmed that SRGN was heavily decorated with long chains of CS, and to a lesser degree of HS (Fig. 2e). Using antibodies specifically recognizing the individual sulfated disaccharide stubs at the non-reducing termini of GAG chains after enzymatic digestion revealed that SRGN GAG contained both CS (CΔ4S and CΔ6S) and HS-derived disaccharide covalently linked to the tetrasaccharide linkage region to initiate its GAG chains.

### SRGN-mediated cell migration is dependent on the interaction of its GAG moiety with CD44

**Fig. 3:**
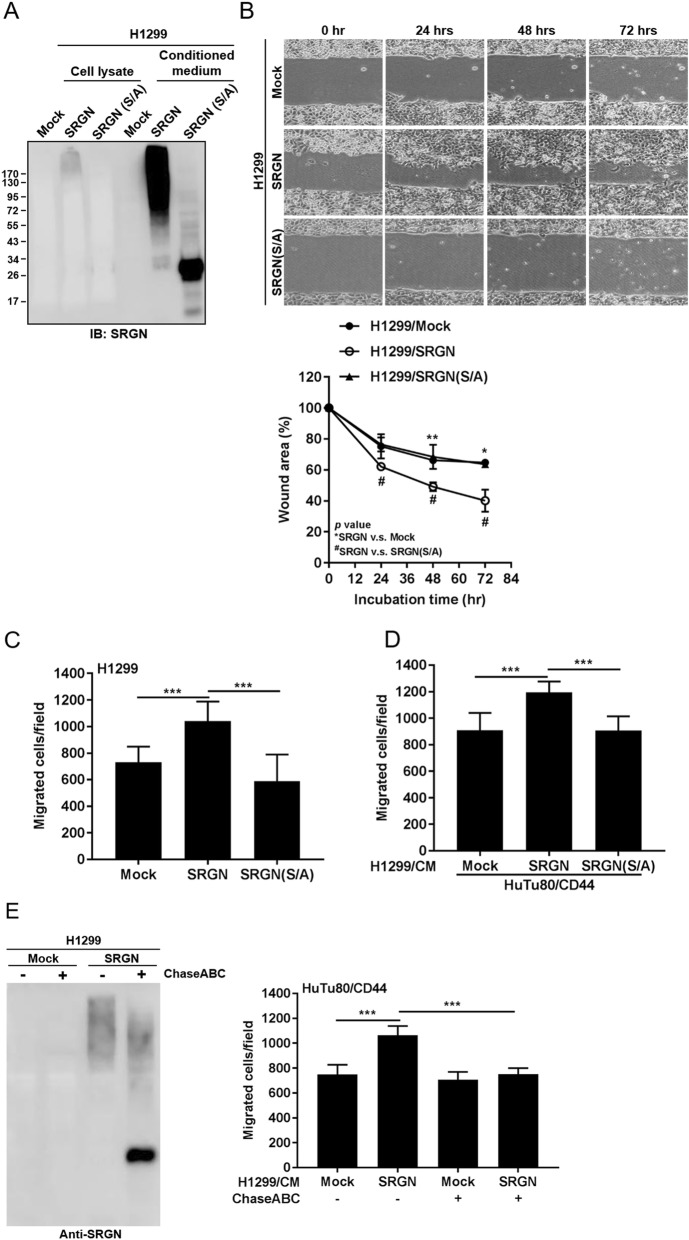
CS-GAG modification is critical for SRGN-mediated cell migration. **a** Western blot analysis of SRGN in the total cell lysate and CM of H1299/Mock, H1299/SRGN and H1299/SRGN(S/A) cells cultured in serum-free medium for 48 h. **b** Wound healing assay was performed in H1299/Mock, H1299/SRGN and H1299/SRGN(S/A) cells. After 72 h, wound area (lower panel) was assessed by ImageJ and normalized to 0 h. **c** Boyden chamber migration assay was performed in H1299/Mock, H1299/SRGN and H1299/SRGN(S/A) cells. Migrated cells were counted in 3 h. **d** Boyden chamber migration assay was performed in HuTu80/CD44 cells that were suspended in CM derived from H1299/Mock, H1299/SRGN or H1299/SRGN(S/A) cells, respectively. Migrated cells were counted in 5 h. **e** CM harvested from H1299/Mock and H1299/SRGN cells were treated with or without ChaseABC for 24 h, and subjected to western blot analysis of SRGN. HuTu80/CD44 cells were suspended in non-treated or ChaseABC-treated CM and subjected to Boyden chamber migration assay. Migrated cells were counted in 5 h. Data are presented as the mean ± SD of three independent experiments. *P < 0.05, **P < 0.01, ***P < 0.001 by Student’s *t*-test

In MCF7 breast cancer cells, overexpression of the full-length SRGN (158 amino acids) promoted cell proliferation and migration, however, overexpression of a truncated mutant (containing the N-terminal 111 amino acids) in which the GAG attachment sites were removed failed to promote cell proliferation and migration [22], suggesting a potential role of GAG modification in SRGN-mediated functions. To address whether GAG modification is a prerequisite for SRGN-elicited function, we generated a SRGN(S/A) mutant, in which the eight serine residues in the “serine-glycine” repeats serving for GAG attachment were converted to alanine. The SRGN(S/A) mutant protein was expressed with a molecular weight near that of the core protein (Fig. 3a). Furthermore, in analogy to the GAG-decorated SRGN, SRGN(S/A) protein was readily secreted to the conditioned medium (Fig. 3a). Most importantly, overexpression of SRGN(S/A) mutant that lacked GAG modification failed to promote wound healing as well as cell migration as the intact wild type SRGN did (Fig. 3b and c). In agreement, the conditioned medium containing the SRGN(S/A) failed to promote the migration in CD44-expressing HuTu80 cells (Fig. 3d). Furthermore, chondroitinase ABC digestion of the conditioned medium of H1299/SRGN cells not only converted the high molecular weight SRGN to the size of its core protein, but also abrogated SRGN-elicited cell migration in HuTu80/CD44 cells (Fig. 3e), showing that CS-GAG modification is critical for SRGN-mediated cell migration.

**Fig. 4:**
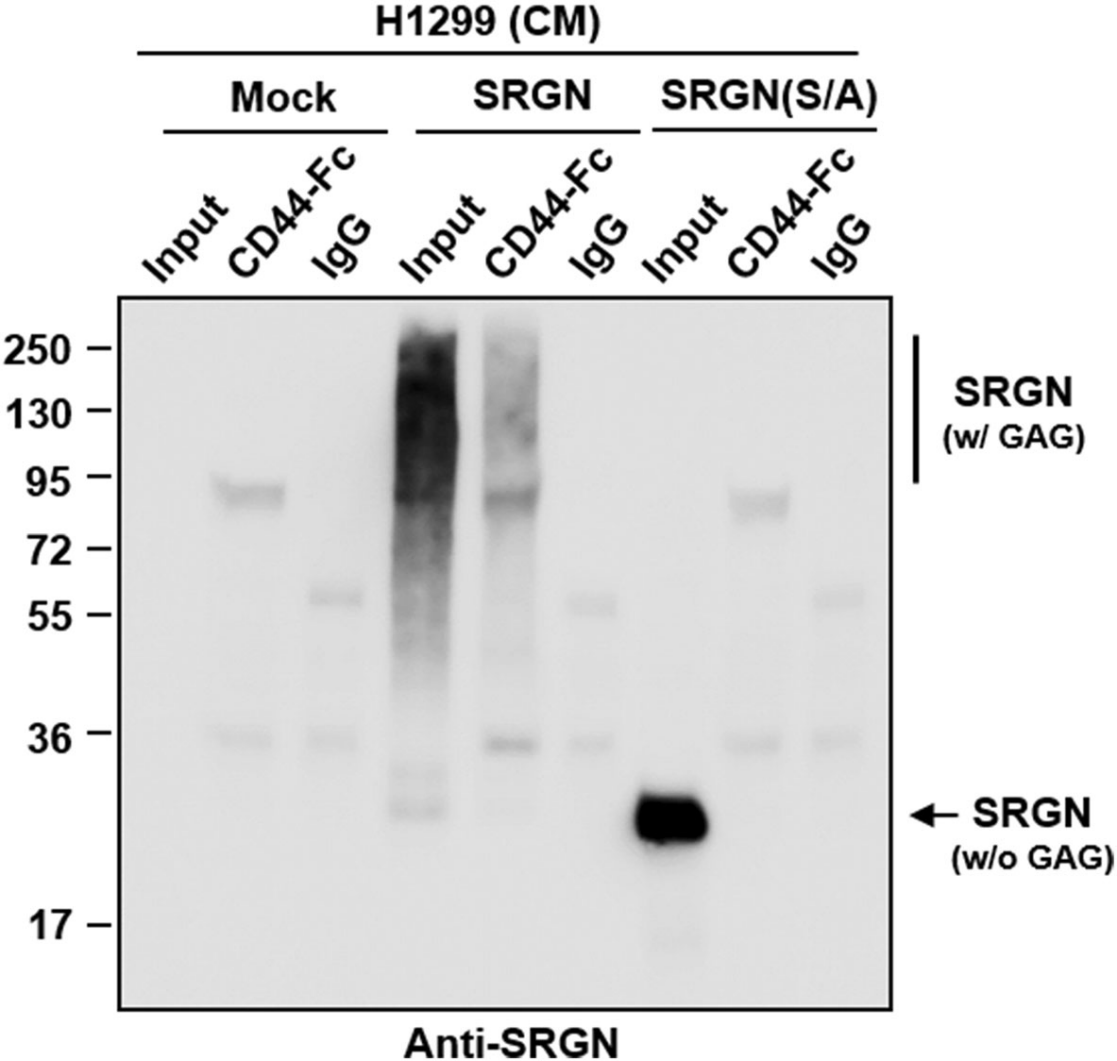
GAG modification is critical for SRGN binding to CD44. CM was harvested from H1299/Mock, H1299/SRGN or H1299/SRGN(S/A) cells, and incubated with CD44-Fc or IgG control for 18 h at 37 °C. Western blot analysis of SRGN in the input proteins and proteins bound to CD44-Fc and IgG using anti-SRGN antibody was shown

In T lymphocytes, SRGN has been shown to interact with cell surface receptor CD44 and promotes lymphoid cells adherence and granzyme A secretion [10]. Removal of CS modification by chondroitinase ABC treatment led to the loss of CD44 interaction [24]. To demonstrate the role of GAG modification involved in CD44 binding, conditioned medium was collected from H1299/SRGN and H1299/SRGN(S/A) cells, and incubated with recombinant human CD44-Fc chimera protein (CD44-Fc) which carries the extracellular domain of CD44. As shown in Fig. 4, the intact GAG-modified SRGN was captured by immobilized CD44-Fc, whereas the SRGN(S/A) mutant devoid of GAG modification was not. These data suggest that GAG modification is critical for SRGN binding to CD44 and SRGN-mediated functions.

### SRGN promotes cytoskeleton reorganization and Rho-family GTPase activation

**Fig. 5:**
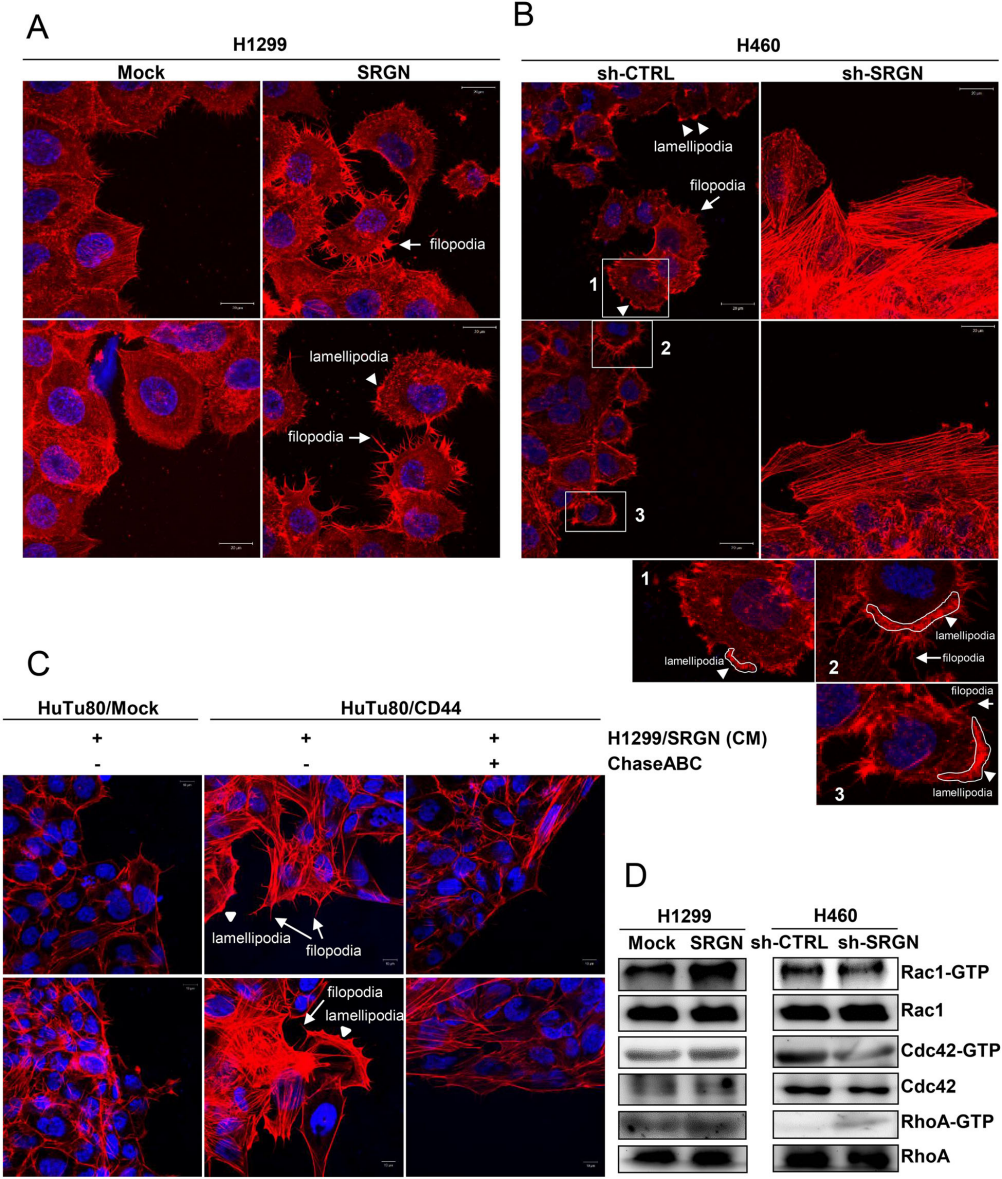
SRGN induces cytoskeleton reorganization and Rho-family GTPase activation. **a-c** Phalloidin staining of actin. H1299/Mock and H1299/SRGN cells (**a**) were incubated in serum-free medium for 24 h and H460/sh-CTRL, and H460/sh-SRGN cells (**b**) were incubated in 2% FBS-containing medium for 72 h, followed by Alexa Fluor™ 594 Phalloidin staining of filamentous actin. **c** HuTu80/Mock and HuTu80/CD44 cells were seeded into the Culture-Insert 2 Well on slide and incubated overnight to form the gap for cell migration. After removing the insert, cells were washed with 1X PBS twice and incubated with CM harvested from H1299/SRGN cells that had been treated with or without ChaseABC for 24 h, followed by Alexa Fluor™ 594 Phalloidin staining of filamentous actin. Filopodia (arrows) and lamellipodia (arrowheads) structures were observed using confocal microscopy. The circumscribed rectangular regions are further enlarged for better viewing. **d** H1299/Mock, H1299/SRGN, H460/sh-CTRL, and H460/sh-SRGN cells were seeded in FN-coated dishes. In 30 min, attached cells were lysed and subjected for pulling down of Rac1-GTP, Cdc42-GTP and RhoA-GTP. Western blot analysis was performed for total and active Rac1, Cdc42, and RhoA as indicated

Since SRGN expression enhances the migratory activity of NSCLC cells, we examined whether SRGN induces actin cytoskeleton re-organization and changes in cell morphology. In H1299 cells, overexpression of SRGN facilitated wound closure, along with significant changes in actin dynamics that numerous filopodia- and lamellipodia-like protrusions were induced (Fig. 5a). On the other hand, knockdown of SRGN expression dramatically delayed wound closure in H460 cells that was associated with an apparent loss of cellular protrusions and concomitant formation of thick stress fibers (Fig. 5b). In consistence to our findings that SRGN promotes cell migration in a CD44-dependent manner, we showed that SRGN-containing medium promoted filopodia- and lamellipodia-like protrusions in HuTu80/CD44 but not in HuTu80/Mock cells (Fig. 5c). Furthermore, removal of CS-GAGs by ChaseABC to block the interaction of SRGN and CD44 suppressed SRGN-elicited cytoskeleton re-organization. Rho-family GTPases are known to modulate cell morphologic change and cell motility through regulating actin cytoskeleton organization [25, 26]. Rac1 activation promotes formation of lamellipodia and active Cdc42 induces filopodia, whereas RhoA activated by lysophosphatidic acid (LPA) promotes the formation of stress fiber [26]. In consistence to our findings that SRGN induced actin cytoskeleton re-organization, SRGN was shown to affect the activities of Rho-family GTPases. Overexpression of SRGN significantly induced Rac1 activation in H1299 cells, and knockdown of SRGN led to decrease in Rac1 and CDC42 activation in H460 cells (Fig. 5d). Along with the induction of stress fiber formation, increased RhoA activity was observed in H460 cells upon knockdown of SRGN. These results support the notion that SRGN induces cytoskeleton re-organization to facilitate cell migration via regulating the activities of small Rho GTPases.

### SRGN promotes cell migration through inducing Src activation and facilitating focal adhesion turnover

**Fig. 6:**
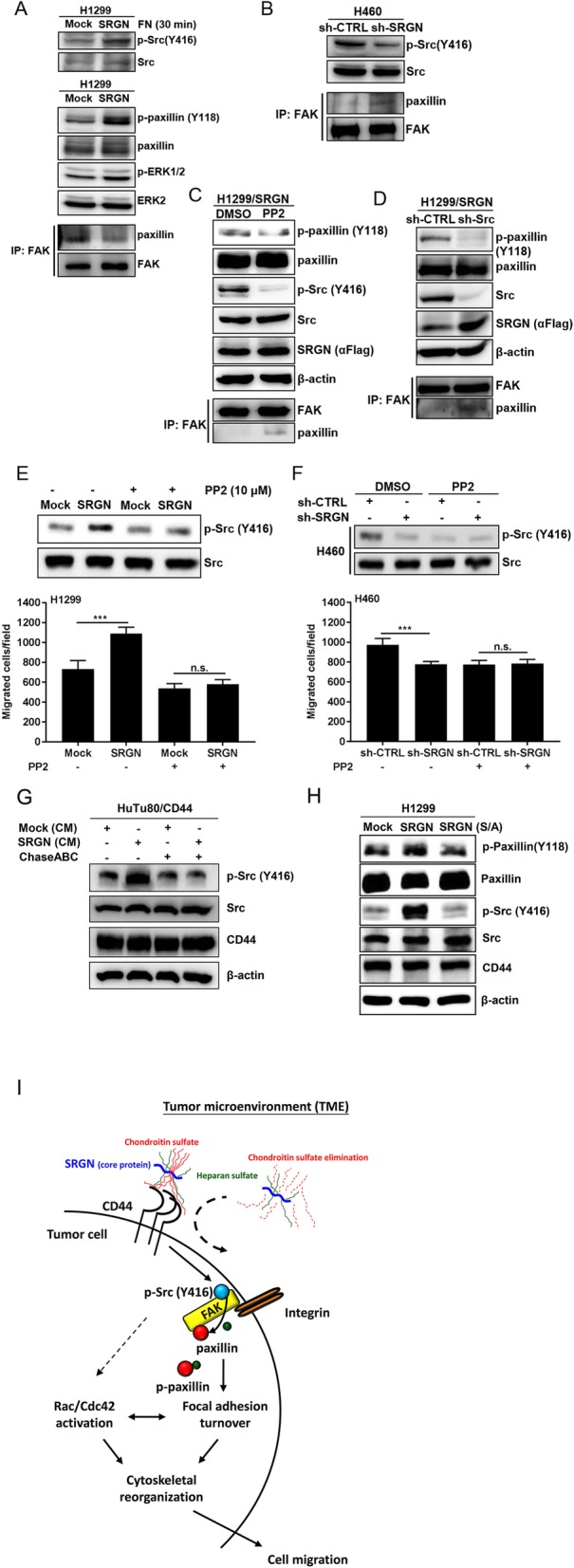
SRGN promotes cell migration through Src-elicited focal adhesion turnover. **a** After serum-starvation for 24 h, H1299/Mock and H1299/SRGN cells were plated in FN-coated dishes. In 30 min, cells were lysed and assayed for Src phosphorylation. In parallel, serum-starved cells were subjected to western blot analysis of phosphorylated paxillin and ERK/MAPK, and co-immunoprecipitation assay for paxillin/FAK adhesion complex formation. **b** H460/sh-CTRL and H460/sh-SRGN cells were cultured in serum-free medium for 24 h and examined for Src phosphorylation and paxillin/FAK complex formation. **c** H1299/SRGN cells were cultured in serum-free medium for 24 h, treated with PP2 (10 μM) or DMSO for 3 h, and phosphorylation of Src and paxillin, and paxillin/FAK complex formation were assessed. **d** Src expression was depleted in H1299/SRGN cells by shRNA-mediated knockdown approach, and phosphorylation of paxillin and the formation of paxillin/FAK complex were assessed in the control-knockdown (sh-CTRL) and Src-knockdown (sh-Src) cells. **e-f** H1299/Mock and H1299/SRGN cells **(e)** or H460/sh-CTRL and H460/sh-SRGN cells **(f)** were treated with or without PP2, and subjected to Boyden chamber migration assay. Migrated cells were counted in 3 h. Western blot analysis of phosphorylated Src and total Src was shown. Data are presented as the mean ± SD of three independent experiments. ***P < 0.001 by Student’s t-test. **g** HuTu80/CD44 cells were incubated with CM harvested from H1299/Mock and H1299/SRGN cells that had been treated with or without ChaseABC for 24 h, followed by western blot analysis of designated proteins. **h** H1299/Mock, H1299/SRGN and H1299/SRGN(S/A) cells were cultured in serum-free medium for 24 h, and subjected to western blot analysis of phosphorylation of Src, paxillin and other proteins as indicated. **i** A working model is proposed for SRGN-mediated cell migration. SRGN in the tumor microenvironment binds to tumor cell surface CD44 via its CS-GAGs, and promotes Src activation and subsequent paxillin phosphorylation and the dissociation of paxillin/FAK adhesion complex, to accelerate focal adhesion turnover. SRGN also promotes the activation of Rac1 and Cdc42 to enhance cytoskeleton reorganization, leading to increased cell migration

Directional cell movement is driven by repetitive cycles of actin cytoskeleton re-organization, including forming protrusions and adhesions at the leading edge, detaching adhesions at the trailing edge, retracting the trailing edge and pulling the whole cell body forward [27]. This process is regulated by synchronous assembly/disassembly of focal adhesion (FA) structures. Increased rate of FA turnover leads to increased cell migration. In growth factor-mediated signaling cascade leading to integrin-mediated adhesions, activated Src phosphorylates integrin-binding protein paxillin, an important scaffold protein, to recruit structural and signaling molecules in FA assembly and disassembly. Modulation of paxillin phosphorylation regulates both the assembly and turnover of adhesion sites [28]. In our study, we showed that SRGN promoted cell migration via its interaction with CD44. Binding of CD44 by its physiological ligand hyaluronic acid (HA) has been reported to promote cell migration via Src activation [14]. In addition, we have previously shown that engagement of CD44 induced lipid raft coalescence to facilitate a CD44-Src-integrin signaling axis [29]. Here, we examined whether Src plays a role in SRGN-elicited NSCLC cell migration by regulating FA dynamics. As shown, SRGN overexpression promoted Src phosphorylation in H1299 cells (Fig. 6a). SRGN overexpression also promoted the levels of phosphorylated paxillin and ERK1/2 (Fig. 6a), both been reported to initiate and mediate the formation of focal complexes and focal adhesions. Increased interaction between FAK and paxillin leading to FA stabilization has been reported to reduce FA turnover and in turn to suppress cell migration [30]. We showed that overexpression of SRGN de-stablized FAK/paxillin interaction in H1299 cells (Fig. 6a), suggesting a role of SRGN in promoting FA turnover. In a reciprocal experiment, we show that knockdown of SRGN in H460 cells reduced the level of phosphorylated Src and promoted stable FAK/paxillin complex formation (Fig. 6b). To demonstrate that Src is a master regulator to mediate SRGN-elicited FA assembly and turnover, we treated the cells with PP2, a selective Src inhibitor, to inhibit Src activity, or performed shRNA-based knockdown approach to eliminate Src transcripts. As shown, PP2 treatment as well as knockdown of Src expression efficiently suppressed paxillin phosphorylation and stabilized FAK/paxillin complex formation in H1299/SRGN cells (Fig. 6c and d). Most importantly, PP2 treatment abrogated SRGN-elicited Src phosphorylation and cell migration in H1299 cells (Fig. 6e). In H460 cells, PP2 treatment exerted similar effects as shRNA-mediated knockdown of SRGN on suppressing Src phosphorylation and cell migration (Fig. 6f). These data suggest that SRGN promotes cell migration in part through facilitating Src-mediated focal adhesion turnover.

To further address that it is the secreted SRGN binding to CD44 to induce Src-mediated focal adhesion turnover, HuTu80/CD44 cells were treated with Mock-CM and SRGN-containing CM. As shown in Fig. 6g, SRGN-containing CM, but not the Mock-CM, induced Src phosphorylation, and the treatment of SRGN-containing medium with ChaseABC to strip off GAGs completely abolished SRGN-mediated Src activation. These data were consistent to our findings that SRGN (S/A) mutant, that is deficient in GAG modification, lost the activity to interact with CD44 and failed to promote cell migration. In support, SRGN (S/A) mutant failed to induce phosphorylation of Src and paxillin (Fig. 6h). These data further substantiated the roles of SRGN in TME in promoting tumor aggressiveness and the importance of CS-GAGs in SRGN-elicited cell migration.

In summary, we provide a working model for SRGN-elicited tumor cell migration (Fig. 6i). In a subset of NSCLC cells, SRGN is overexpressed and readily secreted to the tumor microenvironment. SRGN binds to tumor cell surface receptor CD44 and induces Src-mediated focal adhesion turnover and Rho-family GTPase-mediated cytoskeletal reorganization, leading to increased cell migration. Notably, CS-GAG modification is a prerequisite for SRGN to interact with CD44 and therefore critical for SRGN-mediated function. Our findings highlight an important role of glycosylation in disease manifestation.

## Discussion

Tumor microenvironment (TME) plays an active role in promoting tumor progression. We have previously reported that proteoglycan SRGN was overexpressed in NSCLCs and its overexpression was correlated to poor prognosis [11]. SRGN expressed by carcinoma and the stromal cells was readily secreted to the TME, and SRGN binds to tumor cell surface receptor CD44 and activates downstream signaling pathways, promoting malignant phenotypes. In this study, we dissect the molecular mechanisms underlying SRGN-elicited cell migration. We show that SRGN expressed by NSCLC cells was heavily glycosylated through covalent linkage to CS-GAG chains. Through its GAG moiety, SRGN interacted with CD44 and promoted cell migration via accelerating Src-elicited focal adhesion turnover and cytoskeleton re-organization.

SRGN was initially identified as a granule PG helping in proteases storage and required for maturation of mast cells and cytotoxic T lymphocytes (CTLs) [1, 18, 31]. SRGN was later found to be constitutively secreted by macrophages and endothelial cells [20, 32], and involved in regulating the secretion of chemokines and macrophage migration, respectively. SRGN has recently been shown to be secreted by malignant cells, including multiple myeloma cells, nasopharyngeal carcinoma and NSCLC [11, 19, 21], and functioned in cell adhesion and cell migration. These findings demonstrate that SRGN, expressed by various cell types, are expressed and localized to different subcellular compartments and exerts distinct functions. In addition, SRGN expressed in different cell types are shown to display structural heterogeneity due to the differences in its GAG components and the type and extent of its sulfation [9]. We propose that the structural diversity of SRGN may affects its binding to various target proteins in different subcellular compartments and contributes to the observed functional heterogeneity.

In this study, we showed that SRGN was overexpressed and readily secreted by NSCLC cells in a heavily glycosylated form. By several approaches, we demonstrated that the GAG moiety is required for SRGN-mediated function. First, by cell sorting and CD44 knockout experiments, we showed that CD44 is required for SRGN-mediated cell migration. Next, we generated and expressed the full-length SRGN core protein that was devoid of GAG modification by site-directed mutagenesis and demonstrated that the GAG-deficient SRGN not only had lost CD44 binding activity, but also failed to promote cell migration. Using LC-tandem MS analysis, we further showed that SRGN expressed by NSCLC cells were decorated predominantly by CS-GAGs, and to a lesser degree by HS-GAGs. In support, elimination of CS-GAGs by chondroitinases ABC showed that chondroitinase-sensitive GAGs were important for SRGN-elicited migratory activity.

SRGN in bone marrow mast cells is heavily decorated by heparin and required for storage of proteases [31]. Interestingly, the storage defects seen in SRGN−/− mast cells were similar to those seen in mast cells with loss of N-deacetylase/N-sulfotransferase 2, the enzyme critical for heparin sulfation [33, 34]. These findings suggest a possible role of negatively charged SRGN GAG chains in association with various proteases, cytokine and growth factors to regulate multiple immune functions. In cytotoxic T lymphocytes, SRGN has been reported to interact with CD44 for peripheral lymphocyte adhesion and granzyme A release. This ligand-receptor interaction is dependent on the CS-GAG of SRGN [10]. Other than HA, CD44 has been demonstrated to interact with CS [10, 35, 36]. CD44 has been shown to bind to SRGN and HA with high affinity, and bind weakly to C4S, C6S, DS, HS, and heparin [10]. The different binding activity suggests a role of the negative charges of these ligands in the binding to CD44. We show that SRGN interacted with a recombinant human CD44-Fc chimeric protein that contained CD44 extracellular domain (amino acids 22–220). HA binding to CD44 was shown to be competed by increasing concentrations of SRGN [10], suggesting these two ligands may bind to CD44 at similar or overlapping sites and that SRGN may exert higher affinity than HA towards CD44 binding. HA is a long chain, high molecular weight negatively charged polysaccharide and SRGN contains long chains of sulfated GAG with stronger negative charges. It is possible that CD44 interacts with these ligands through electrostatic interactions, and therefore SRGN displays higher affinity than HA towards binding to CD44. It remains to be tested whether CD44 binds to these GAG ligands through electrostatic interaction and the presence of multiple long arrays of negative charges may allow the clustering and activation of CD44. It also remains to be investigated whether other secreted CS-PGs with multiple GAG chains, such as aggrecan and versican may directly interact with CD44 and activate CD44 downstream signaling. Other than SRGN and HA, CD44 can also interact with various components in the extracellular matrix, including collagen, lamanin and fibronectin, and such interaction may promote cell adhesion and migration [37]. As shown in Fig. 1e, KD of SRGN inhibited cell migration, and knockout of CD44 inhibited cell migration to a much greater degree (Fig. 1e). We conclude that SRGN binds to CD44 efficiently, and in part promotes CD44-mediated cell migration.

Increasing evidence has shown that the heterogeneity of CS composition, namely the degree of polymerization and sulfation modification, may affect its functions at the cellular level. We show that SRGN expressed by H1299 and H460 cells were decorated predominantly by CS-GAGs, and that SRGN expression conferred migratory activity to both cells, despite the fact that SRGN GAGs in these two cell lines varied greatly in the extents of CS disaccharides bearing sulfation at C4- or C6-positions of N-acetylgalactosamine. We noted that CM harvested from H1299/SRGN cells contained a significant amount of HS GAG, however, the HS GAG was mostly composed of non-sulfated HS disaccharides. As to the comparative studies to assess the amounts of GAGs and disaccharides in the CM derived from H1299/Mock versus H1299/SRGN cells and H460/sh-CTRL versus H460/sh-SRGN cells, the differential amounts associated with SRGN expression in these two NSCLC cells may be indicative of the relative amounts of GAGs and disaccharides associated with SRGN, but may also contain the components derived from other proteoglycans whose expression is regulated by SRGN expression.

CD44 has been reported to serve as a platform for signaling transduction, including MAPK/PI3K and β-catenin signaling, to promote tumor progression and metastases [38]. By RT-PCR analysis, we showed that H460 cells, in comparison to H1299 cells, expressed *SRGN* at a significantly higher level, along with elevated expression of CS synthesis and chain elongation enzymes, including chondroitin sulfate N-acetylgalactosaminyltransferase 1 (*CSGALNACT1*), chondroitin sulfate synthase 3 (*CHSY3*), chondroitin polymerizing factor (*CHPF* and *CHPF2*). In consistence, higher amounts of SRGN-derived GAGs were harvested from the CM of H460 cells. In consistence to the findings reported in NPC cells that extracellular matrix SRGN has recently been shown to upregulate the expression of CD44 in an autocrine manner via reciprocally activating the MAPK/β-catenin axis [39], we also observed that SRGN KD significantly downregulated CD44 and nuclear β-catenin level in H460 cells (data not shown). CS-GAGs rich in chondroitin sulfate E (CS-E) disaccharide unit was shown to bind Wnt3A tightly, whereas other CS isoforms and HS showed little binding activity [40]. Exogenous CS-E also showed potent inhibition on Wint3A-induced beta-catenin accumulation [40], and application of CS-E negatively regulated breast cancer cells motility by interfering Wnt signaling [41]. By LC-tandem MS analysis, we showed that SRGN derived from H460 cells was modified by GAGs with a high amount of CS-E disaccharide unit, which was in agreement to the high level of carbohydrate sulfotransferase 15 (*CHST15*), which encodes GalNAc 4-sulfate 6-O-sulfotransferase (GalNAc4S-6ST) responsible for transferring sulfate from 3′-phosphoadenosine 5′-phosphosulfate (PAPS) to the C-6 hydroxyl group of the GalNAc 4-sulfate residue of chondroitin sulfate A (CS-A) to yield CS-E [4]. Notably, CD44 can interact with LRP6 and functions as a co-receptor for Wnt signaling transduction [42]. It remains to be further investigated whether the ECM SRGN derived from H460 cells may help in recruiting growth factors and ligands through CS-E-enriched GAG chains and regulate pro- or anti-tumorigenic signaling, such as WNT/β-catenin pathway, by CD44 platform in cancer progression.

Interfering CD44-ligand interaction, such as HA binding, has been reported as a potential strategy to suppress tumor metastasis and recurrence [43–45]. Interestingly, A6, a peptide sharing sequence homology with HA binding domain of CD44, strongly enhanced the adherence of CD44-expressing cancer cells to HA, and effectively suppressed tumor cell migration in vitro and tumor metastasis in xenografted B16-F10 mouse model [46]. SRGN has been shown to promote cell migration in several types of cancer, and SRGN promoted NSCLC cell migration in a CD44-dependent manner. Although SRGN binding region is located near to HA-binding domain, the molecular mechanism underlying SRGN-mediated cell migration is still unclear. HA binding to CD44 has been shown to promote ovarian cancer cell migration by recruiting Src and promoting cortactin-mediated cytoskeleton function [14]. In this study, we show that SRGN promoted NSCLC cell migration mediated through CD44-elicited Src phosphorylation and Src-mediated phosphorylation of paxillin. Phosphorylation of paxillin, an ERK-regulated scaffold, has been shown to induce subsequent signaling cascades to coordinate FAK and Rac activation in the vicinity of focal adhesions, thus promoting the rapid focal adhesion turnover and lamellipodia extension to accelerate cell migration [47, 48]. In the present study, we show that SRGN-induced Src phosphorylation, leading to paxillin phosphorylation and disassembly of FAK/paxillin complex formation, resulting increased focal adhesion turnover. In parallel, in accordance to previous reports that CD44 regulates Rho-family of GTPases to promote tumor progression [49–53], we show that SRGN promoted cytoskeleton reorganization via inducing Rac1 and CDC42 activation. Src tyrosine kinase has been reported to play an important role in cell adhesion and spreading, and crosstalk between growth factor receptor, e.g. EGFR, and integrins also regulates Src activation to control Rho-GTPases [54]. It remains to be investigated whether SRGN/CD44 interaction-induced Rho-family GTPase activation is mediated through Src activity. In addition to lung cancer, SRGN has also been reported to promote aggressive phenotypes in breast cancer cells by inducing epithelial-mesenchymal transition, and up-regulating the production of proteolytic activity and proinflammatory cytokines, including IL-8 [55]. SRGN was shown to promote the expression and secretion of IL-8, which in turn promoted cell proliferation, migration and invasion via IL-8/CXCR2 signaling axis to trigger several downstream pro-survival and pro-migratory pathways, including PI3K, Rac and Src signaling. CD44 has also been reported to interact with intermediate molecular mass HA to enhance neutrophil phagocytosis and IL-8 production via p38 and ERK1/2-MAPK signaling pathways [56]. Taken together, these findings suggested that tumor cell surface receptor CD44 plays an important role in responding to tumor microenvironment-associated ligands, including SRGN, to potentiate cancer cell aggressiveness via triggering cytoskeleton remodeling and pro-survival pathway.

In summary, we provide a working model of SRGN-elicited cell migration. SRGN secretory protein binds to CD44 via its CS-GAG chains. The SRGN/CD44 axis promotes Src activation and paxillin phosphorylation to facilitate FA turnover. Moreover, SRGN also promotes RAC1 and CDC42 activation to induce the formation of filopodia and lamellipodia of migrating cells. Notably, removal of CS-GAG chains efficiently suppresses SRGN-elicited cell migration, suggesting that targeting specific glycans on tumor-derived ligands in TME may be a potential strategy for cancer therapy.

## Conclusions

We demonstrate that proteoglycan SRGN is readily secreted to the extracellular matrix in NSCLC cells in a glycosylated form heavily decorated with CS-GAGs. SRGN in the tumor microenvironment binds to tumor cell surface CD44 via its GAGs, and facilitates cytoskeleton reorganization and Src-mediated focal adhesion turnover, leading to increased cell migration. Elimination of CS blocks SRGN-elicited cell migration, and therefore provides an attractive strategy by targeting SRGN for CD44-expressing NSCLC.

## Supplementary information

**Additional file 1:** **Figure S1.** H1299/Mock and h1299/SRGN cells as well as H460/Sh-CTRL and H460/Sh-SRGN cells were cultured in serum-free medium for 48 h. SRGN expression in whole cell lysate (WCL) and conditional medium (CM) was assessed by western blot analysis.

**Additional file 2:** **Figure S2.** Quantitation of SRGN-related GAGs and GAG disaccharide components in NSCLC cells. **a** LC-MS/MS profiles of HS and CS disaccharide standards. **b** LC-MS/MS profiles of HS and CS disaccharides derived from the CM of H1299/SRGN and H460/sh-CTRL cells.

## Data Availability

All materials are available by the corresponding author.

## Abbreviations

Chase: Chondroitinase
CS: Chondroitin sulfate
GAG: Glycosaminoglycan
HS: Heparin sulfate
SRGN: Serglycin
