# Supplementary figures and images for: Proteoglycan serglycin promotes non-small cell lung cancer cell migration through the interaction of its glycosaminoglycans with CD44
Source: J Biomed Sci. 2020 Jan 2;27:2. doi: 10.1186/s12929-019-0600-3 (PMC6939340; doi:10.1186/s12929-019-0600-3)

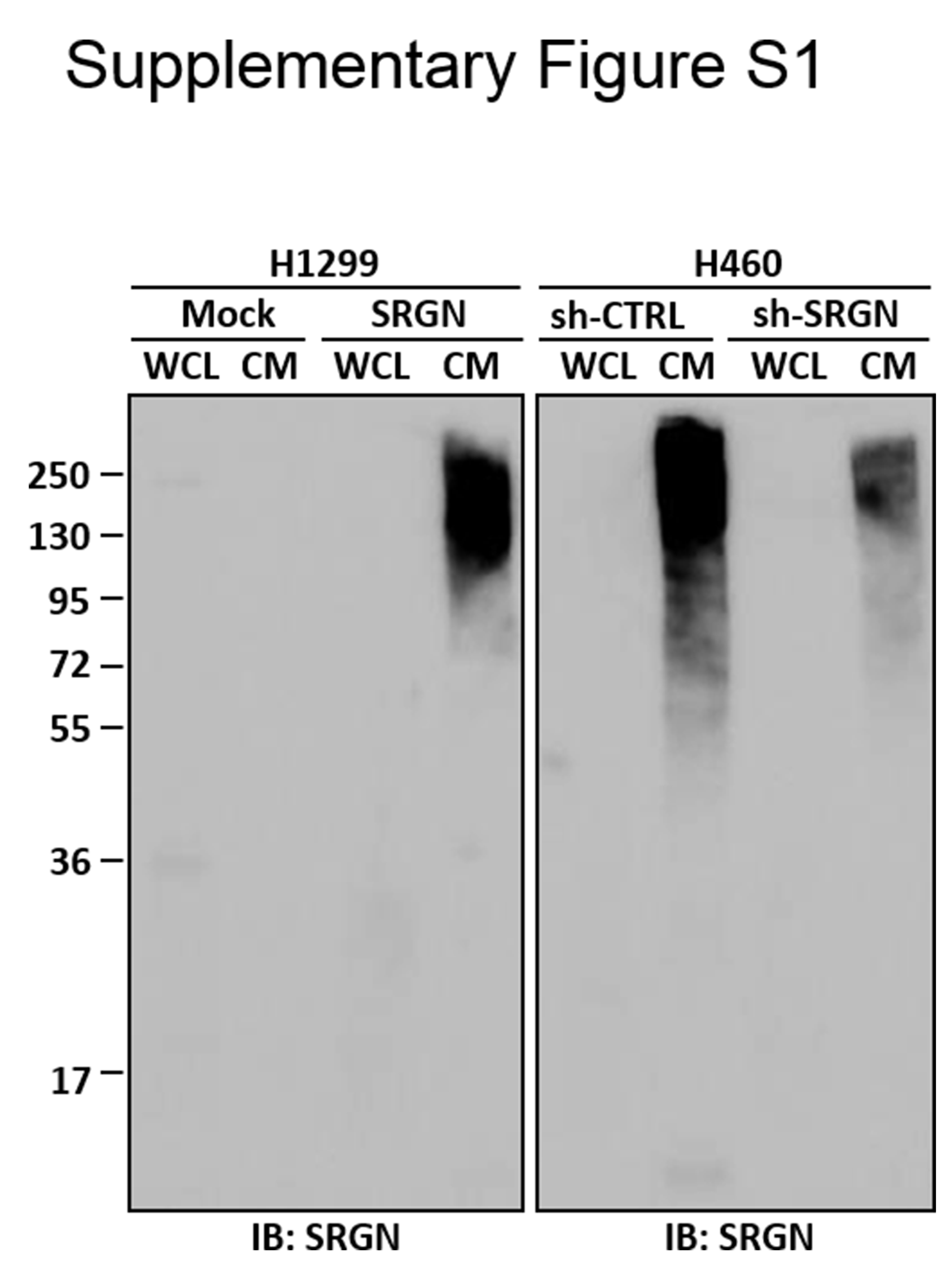

Supplement: Supplementary file 1 — Additional file 1: Figure S1. H1299/Mock and h1299/SRGN cells as well as H460/Sh-CTRL and H460/Sh-SRGN cells were cultured in serum-free medium for 48 h. SRGN expression in whole cell lysate (WCL) and conditional medium (CM) was assessed by western blot analysis. [file 12929_2019_600_MOESM1_ESM.jpg]

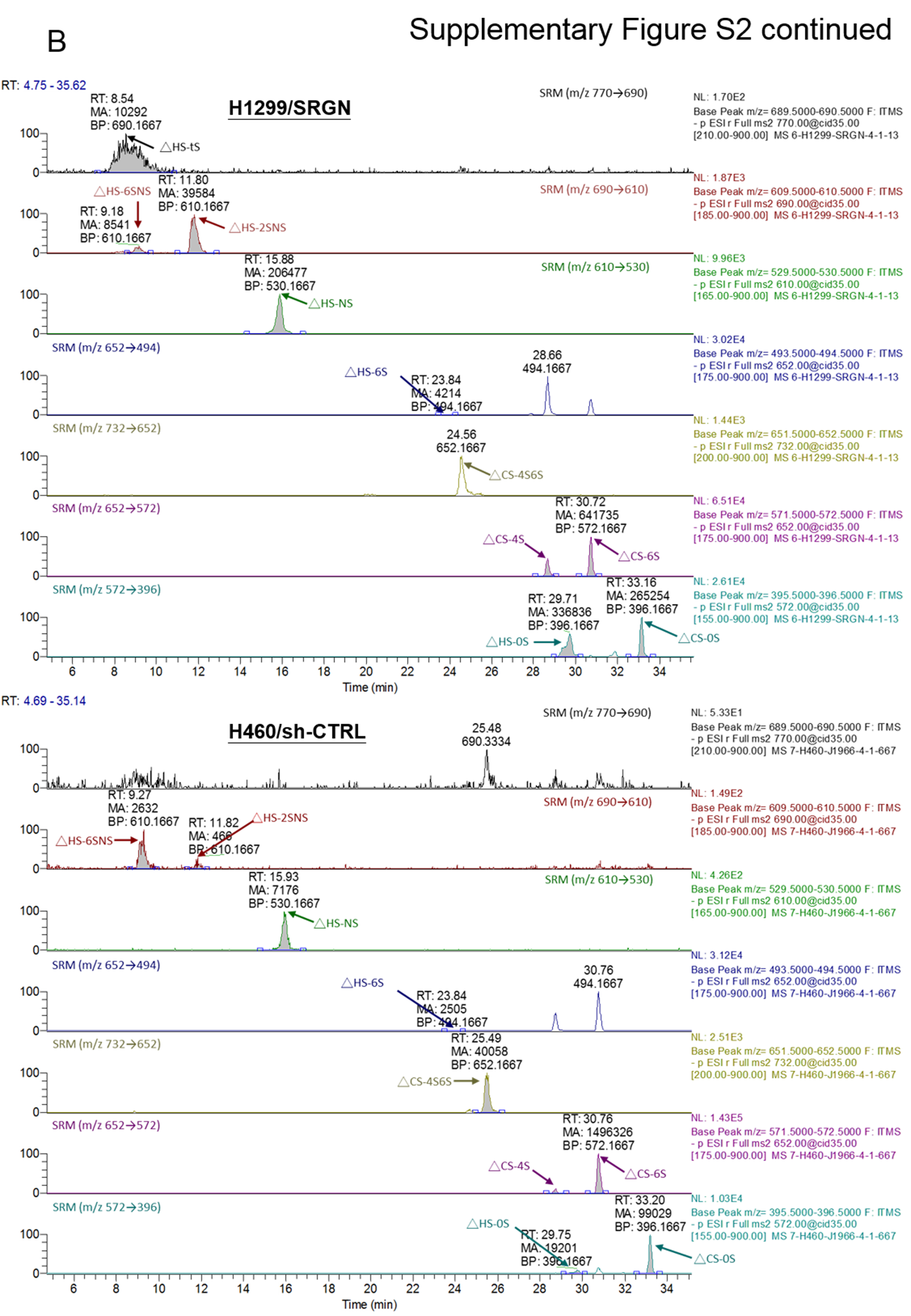

Supplement: Supplementary file 2 — Additional file 2: Figure S2. Quantitation of SRGN-related GAGs and GAG disaccharide components in NSCLC cells. a LC-MS/MS profiles of HS and CS disaccharide standards. b LC-MS/MS profiles of HS and CS disaccharides derived from the CM of H1299/SRGN and H460/sh-CTRL cells. [file 12929_2019_600_MOESM2_ESM.zip › Fig.S2 continued.jpg]

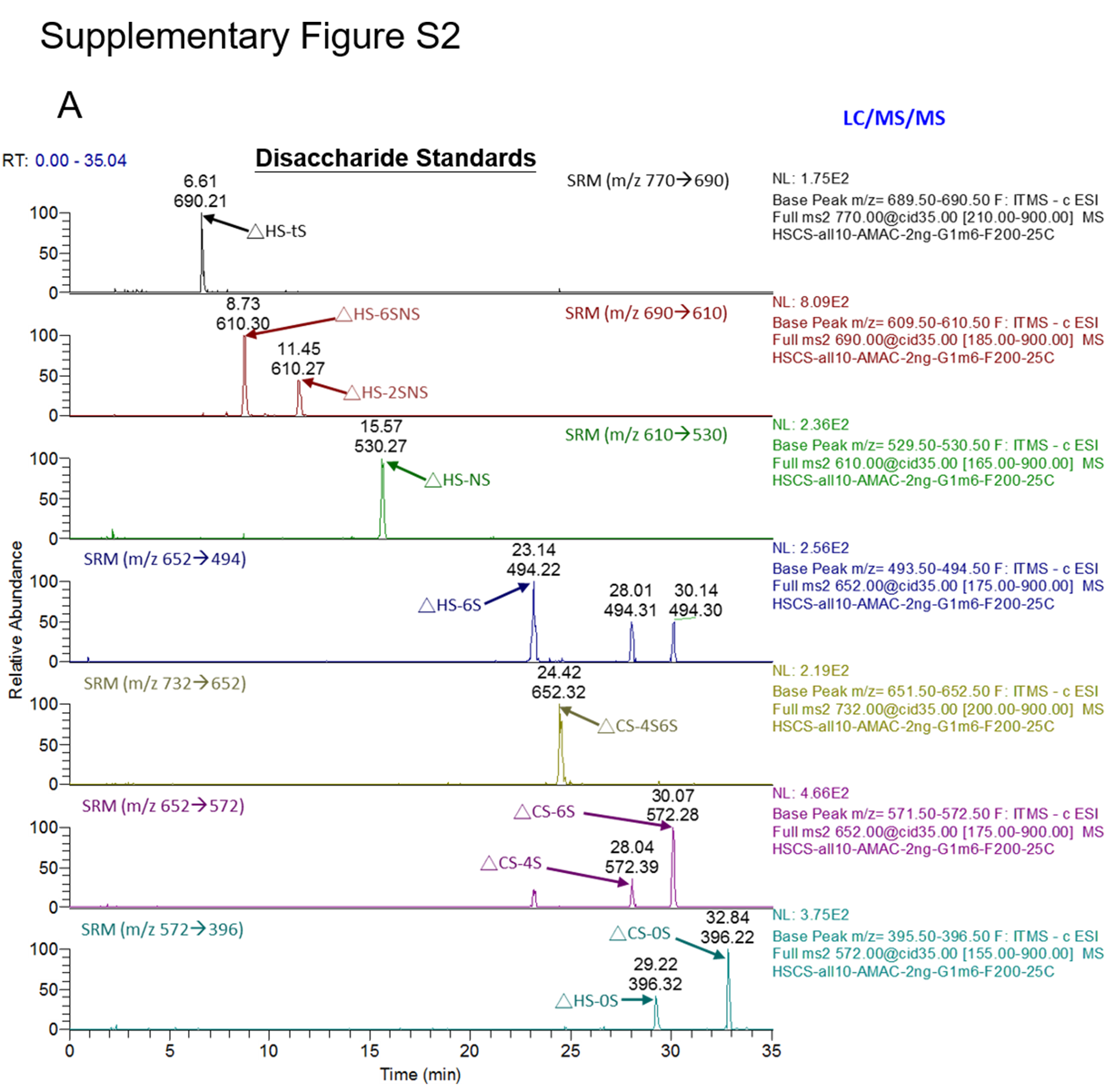

Supplement: Supplementary file 2 — Additional file 2: Figure S2. Quantitation of SRGN-related GAGs and GAG disaccharide components in NSCLC cells. a LC-MS/MS profiles of HS and CS disaccharide standards. b LC-MS/MS profiles of HS and CS disaccharides derived from the CM of H1299/SRGN and H460/sh-CTRL cells. [file 12929_2019_600_MOESM2_ESM.zip › Fig.S2.jpg]
